# Role of periostin in ECRS

**DOI:** 10.1007/s00405-020-06369-x

**Published:** 2020-09-21

**Authors:** Lei Yu, Jisheng Wang, Kai Liu

**Affiliations:** Weihai Municipal Hospital, Weihai, China

**Keywords:** Periostin, Eosinophilic, Chronic rhinosinusitis

## Abstract

Periostin, an extracelluar matrix protein belonging to the fasciclin family, has been reported to play a key role in the process of Th2-inflammation disease. As eoshinophilic chronic rhinosinusitis has a higher incident rate, studies show that periostin has participated in the process of inflammation and remodeling. This review mainly to summarize researches of periostin in ECRS and to investigate the clinical significance and expression of periostin.

## Introduction

CRS is a heterogeneous disease, with different inflammatory endotypes, each of which appears various pathophysiologic mechanisms. Eosinophilic chronic rhinosinusitis (ECRS) is a subgroup of chronic rhinosinusitis, characterized by eosinophilic infiltration and tissue remodeling, driven by Th2 responses, which is always accompanied with high levels of Th2 related cytokines. Notably, the incidence rate of ECRS has gradually became higher than the past, either in western countries or east Asia. For clinical treatments, it is urge to provide effective and personalized for ECRS patients, especially with severe inflammation, in face of quite large proportions of those with poor outcomes.

### Biology of periostin

Periostin is a 90-kDa member, belonging to the fasciclin-containing family, and in human beings, periostin genes have been found to locate in 13q13.3 and the length of cDNA is 3187 bp [1–3]. Takeshita S first found periostin in 1993 from a mouse osteoblast cell line and as a secreted factor, it was originally termed osteoblast-specific factor 2 [4].

As advanced studies with periostin, it has been known to play a great role not only in wound repair, heart morphogenesis, metastases, and cancers [5–7], but also in eosinophil-associated with inflammatory disease including upper airways and lower airways, such as bronchial asthma and ECRS [8, 9].

Additionally, periostin has been thought to be a extracelluar matrix protein secreted by connective tissue cells and fibroblasts, and located in mucosa of upper airway disease, lower respiratory and myocardium [10]. It has been proved that periostin can take part in collagen formation, fibrosis, epithelial mesenchymal transition, tumor development, Th2-driven inflammation and a variety of injury-repair mechanisms.

Besides many previous studies, periostin has made contribution to the development and maintaince of inflammatory diseases, such as bronchial asthma, allergic rhinitis and chronic rhinosinusitis, aspirin-exacerbated respiratory diseases, allergic bronchopulmonary aspergillosis, and eosinophilic granulomatosis with polyangiitis [3, 11] via different pathways. For example, the role of periostin in bronchial asthma had been proven to play an important role as an extracellular matrix protein in subepithelial fibrosis [12] and airway remodeling [13]. Serum POSTN has been proven to increase in inflammatory diseases such as idiopathic pulmonary fibrosis [14] and stopic kerato conjuctivitis [15]. To achieve this function mainly because a variety of cytokines could upregulate or downregulate the periostin production, its production is controlled by IL4, IL-13, IL-5, TGF-CCN/CTGF (connective tissue growth factor), mechanical stretch, cancer-derived factors angiotensin II, and BMP-2 (bone morphogenetic protein 2) [16–22]. The periostin expression could be induced by transforming growth factor-β, which has dramatic effects on periosteal expansion. Moreover, IL-4 and IL-13 could induce the production of periostin in airway epithelial cells. Based on known knowledge, there are three kinds of tissue-resident cells such as fibroblasts, epithelial cells, and endothelial cells, known to express periostin protein by stimulating IL-4 or IL-13. Previous publications demonstrated that inhibition or downregulation of STAT6 could reduce periostin expression, which correlated with IL-13 and IL-4 secretion, meaning that STAT6 activation is critical [23].

### Periostin in related otolaryngological and eosinophilic diseases

With the sequent studies of periostin, there is no deny that periostin has been closely associated with eosinophilic disease. In the follow part, we will summarize periostin in some kinds of related diseases. Ohta et al. [24] explored the role of periostin in eosinophilic otitis media (EOM) and found that periostin was strongly expressed both in the basement membranes and granulation tissues. Similarly, in vitro animal models of EOM, they found middle ear tissues also appeared periostin immunoreactivity [25].

In eosinophilic esophagitis (EOE), compared with control groups, they found there was overexpression of periostin in esophageal tissues obtained from EOE patients. In the part of allergic rhinitis (AR), in comparison with normal nasal tissues, periostin levels significantly increased in the basement membranes of AR patients [26]. Lately, more and more people prefer to suggest that periostin can involve in processes of Th2-dominance diseases including inflammation and remodeling. Moreover, periostin has played a great role not only in AR, but also in CRS, which including various inflammatory endotypes according to EPOS2020 [27]. Given above studies, we could conclude periostin mainly secrets in some subgroups of CRS, especially in ECRS, high level of IL-5, AFRS and cormibid with asthma or allergic rhinitis.

In this review, we mainly integrate the available findings on periostin and its roles in ECRS as follows to better explore mechanisms and provide personalized therapies.

## Periostin in ECRS

ECRS impairs the quality of life affected persons and is correlated with substantial medical cost. The heterogeneity of ECRS is now widely recognized, encompassing various pathogenic factors and inflammatory mechanisms, yet some of which still is unknown. According to EPOS2020 [27], ECRS is defined as > 5 or > 10 eosinophilis per high-power field (HPF), and has been characterized by eosinophilic dominant inflammation. Genome-wide transcription studies demonstrated that nasal polyps showed remarkedly higher gene expression of periostin as compared with normal sinus mucosa and lately, real-time quantitative reserve transcription polypmerase chain reaction (PCR) and immunohistochemistry (IHC) also confirmed this [28]. It has been reported that ECRS patients showed high mean serum levels of POSTN in comparison with the control group, and ECRSwNP patients has increased levels versus ECRSsNP patients [29, 30]. In comparison with non-ECRSwNP, ECRSwNP have increased periostin mRNA and periostin levels [31]. As is known to us, periostin expression shows high in ECRS compared with control. Stankovic et al. first published the existence of periostin in intact nasal mucosa and polyp tissues [28]. As classified standards for CRS has developed, people tests periostin levels in different CRS endotypes combined with advanced criteria.

Some scholars examined periostin levels in CRS and results showed that periostin levels were higher in ECRS patients than other various subgroups, meaning ECRS may have an unkown association with periostin [32] and Ishida et al. also found similar results [26]. In mRNA levels, Kim et al. [31] found that periostin mRNA levels are significantly increased in ECRS than in non-ECRS.

But we must admit the fact, in early time, the standard for ECRS was not so strict and accurate without considering whether accompanied with asthma.

Later, Asano teams [33] tested periostin expressions in ECRS with asthma and without asthma, respectively, and found the former showed high results compared with the latter, which indicating ECRS with other kinds inflammatory disease may significantly accelerate periostin expression. Some people provoked the idea that ECRS had a unique mechanism. Based on reported cases [34], we can detect that serum expression of periostin has no association with asthma severity, but relates to ECRS severity.

In following part, we summarize periostin in ECRS in current from three viewpoints: tissue expression, serum expression and location.

From tissue expression, in vitro mouse models, Sang-Wook teams [35] investigated the relationship between periostin expression and polyp lesion formation and infiltration and found the role of periostin might appear a protective in the development of ECRS. Xu et al. [36] investigated protein and mRNA levels of periostin and VEGF in tissues from ECRSwNP, non-ECRSwNP and control groups, indicating that in ECRSwNP patients there were markedly higher in ECRSwNP than other two groups.

In the view of serum production, Ninomiya et al. [37] subdiveded CRS patients into four subtypes: non-ECRS, mild-ECRS, moderate ECRS and severe ECRS following the criteria of JESREC (Japanese Epidemiological Survey of Refractory Eosinophilic Chronic Rhinosinisitis) scoring system, and their results indicated there was a positive association between serum periostin level and severity of ECRS. Using ELISA to examine serum periostin level, they found which gradually increased along with the severity of ECRS.

For location, in addition, some people may pay more attention on the place where periostin locates in CRS via IHC, and now it has been widely acknowledged that there are double models: diffuse type and superficial type.

Shiono et al. [38] first reported that in CRS patients there were two periostin patterns: diffuse type, which meant periostin was located throughout the lamina propria starting just below the basement membrane and superficial type, which meant periostin was only expressed in the subepithelial layers between the basement membrane and the nasal gland; meanwhile, they also detected higher eosinophilic count in former than the latter. Combined with above models, Shionno et al. [38, 39] found mucus eosinophils were related to periostin location, and local periostin location followed “diffuse model” in ECRS while that showed “superficial type” in non-ECRS. Furthermore, the location of periostin is also correlated with variable degrees of inflammation. Shionno et al. published that POSTN deposition pattern changed with the number of eosinophils in NPs patients [38], and later, Ninomiya et al. [39] reported that distribution of POSTN has been determined by immunohistochemical analysis and has differences between between the non-ECRS and severe ECRS phenotypes, showing that in health subject, POSTN deposition was observed only in the subepithelial layer, and in CRSwNP from patients in the superficial type. Additionally, they also investigated the diffuse type of POSTN deposition was frequently observed in high-scores subgroups than low-scores subgroups.

Periostin has been proved that it has participated in many parts of ECRS, among which periostin has an important influence in inflammation and remodeling in ECRS. We will summarize these as follows.

### Periostin and ECRS inflammation

Combining with previous studies, periostin has taken part in eosinophilic inflammation in airway, which also has been thought as the biomarker airway inflammation in eosinophilic-asthma, suggesting us that periostins should be considered a special marker in ECRS, because of playing a great role in ECRS. ECRS is a disease characterized by eosinophilic infiltration, in which EOS plays a crucial role. Some publications have confirmed that there is a close relationship between periostin and eosinophilic inflammation.

It has been proven that periostin could participate in different parts in eosinophilic inflammation in ECRS patients.

Regrading the relationship between periostin and ECRS inflammation. Initially, researchers established mouse model and found periostin might play a protective role in the development of eosinophilic chronic rhinosinusitis with nasal polyps [35]. Ishida A found that periostin has an influence on activating eosinophilis and sustaining eosinophil-mediated inflammation [26]. It has been demonstrated that POSTN is correlated with Th2 inflammation [40, 41], some researchers investigated the correlation between serum POSTN and eosinophil percentage, suggesting that there has been positive associations with blood eosinophil percentage and tissue eosinophil infiltration [39].

About periositn in ECRS mechanisms, although there are many studies about CRS inflammatory responses, but yet has a precise conclusion on mechanisms. In current, many people choose from the relationship between inflammation and periostin to explore and establish the mechanism and regulatory network of periostin in ECRS. Moreover, inflammatory cytokines and periostin could mutually affect each other. For example, Kim et al. found IgE stimulation could through integrin binding induce periostin production and activates epithelial cells to secrete thymic stromal lymphopoietin, which in turn induces the production of IL-5 by mast cells [31]. Interestingly, recent publications demonstrate that osteitis may be reckoned as a marker of ECRS, which may due to local or systemic mediator of inflammation periostin. Snidvongs et al. [42] found osteitis was associated with tissue eosinohilia and increased expression of periostin. Osteritic changes has been proven to be related to CRS severity accompanied with a higher presence of bony remodeling, which could be evaluated by CT, but the association between osteitis and periostin in ECRS needs further researches. Finding this link can help better diagnose and proganose ECRS patients. However, Zuo et al. [43] investigated the average ethmoid osteitis index in ECRS patients and non-ECRS patients, resulting in the index of the former was remarkably lower than the latter. This disparity may be caused by various territory, race or environment and lifestyles. However, pathways related to how periostin and EOS mutually affect haven’t yet been known. So far, there have been some possibilities, such as through the αMβ2 [44], forming pedosomes, ADAM8 [45], resulting in EOS adhering to periostin in extracellular matrices. αMβcould intergrate with periostin and take part in epithelial–mesenchymal transition, as a part of ECRS, but in ECRS, this process still is unknown.

### Periostin and ECRS remodeling

Periostin has been proved to play a regulatory role in fibrosis and collagen deposition and formation, epithelial–mesenchymal transition and a variety of repair mechanisms. Initially, large studies about the role in asthmatic epithelial cells were performed, periostin was found to be essential in mucosal remodeling in the lower airway. It has been reported that POSTN itself and this complex consisting of collagen type I, fibronectin, tenascin-C, and POSTN assist in collagen formation, subepithelial fibrosis and tissue remodeling [2, 20]. ECM proteins may potentially to serve as early biomarkers of remodelling changes in nasal and sinus mucosa.

Given these findings, people gradually investigate the periostin in CRS and in further in ECRS. As is known to us that remodeling process is characterized in ECRS, mainly consisting tissue oedema in the sinus mucosa nasal polyps formation, fibrosis, fibrin deposition, basement membrane thickening (BMT) and epithelial barrier abnormalities[32, 46], which always lead to impaired mucosal function and has been thought to cause increased nasal obstruction and clinical symptoms [47]. Via a cross-sectional study, they examined the relationship between mucus remodeling defined by basement membrane thickening (BMT over 7.5 µm) and subepithelial fibrosis and tissue eosinophilia, and their results showed stronger grade of periostin expression was positively correlated with the presence pf BMT, fibrosis and tissue eosinophila in CRS patients [48].

In accordance with above studies, in ECRS patients, high expressions could promote the levels of periostin and simultaneously aggravate tissue remodeling process, which can lead more structure changes, such as polyp formation. This also testifies the clinical phenomenon that ECRS patients always have nasal polyps.

Studies have been performed to find association between related cytokines and markers, and remodeling changes in CRS, in which periostin has been proven to be a biomarker for remodeling, especially in BMT and fibrosis [48]. In support of this change, through experimentation, Yang et al. found that periostin could induce activation of the Src/AKT/mTOR signaling pathway, resulting in tissue remodeling by differentiation of fibroblasts into myofibroblasts and expression of extracellular matrix components. Glucocorticoids ameliorate periostin-induced tissue remodeling in chronic rhinosinusitis with nasal polyps [49]. Vascular endothelial growth factor(VEGF), as a key role in regulating of remodeling in ECRS, mainly consisting of tissue remodeling and an edematous nasal mucosa could be promoted by periostin through the focal adhesion kinase(FAK) or signal-regulated kinase 1/2(ERK1/2) signaling pathways [50]. Upregulated periostin promotes angiogenesis in keloids through activation of the ERK 1/2 and focal adhesion kinasepathways, as well as the upregulated expression of VEGF and angiopoietin [51]. Xu et al. [36] indicated periostin could play a crucial role in tissue remodeling in ECRS through inducing VEGF production, showing that periostin could exert regulatory mechanisms on remodeling by regualting VEGF. Recent publications demonstrated in the pathogenesis of airway remodeling in brochial asthma, periostin interacted with extracellular matrix proteins and promoted subepithelial fibrosis via autocrine or paracrine, working as a downstream signal of IL-4 and IL-13. In addition, from above researches, the location of periostin in ECRS, which displays in subepithelial area, also make contributions on promoting connective effects with other EMC protein and fibrosis and epithelial–mesenchymal transition to promote remodeling.

Periostin could participate in development and maintenance of inflammation in ECRS through multiple mechanisms. Previous studies demonstrates that in CRS features of remodeling vary depending on the type of inflammation [52], suggesting us CRS inflammation and remodeling, these two processes mutually affect each other. With regards to above studies, periostin might not only participated in the processes of inflammation and remodeling, but also implicate in connection between them, but this underling mechanism still unclear. One thing is needed to notice that eosinaphilia has been associated with remodelling changes [42].

Importantly, remodeling of mucosa in ECRS may play a great significant role in persistence and recurrence and has been proven to be association with higher prevalence of co-morbid asthma and aspirin sensitivity.

### Regulation of periostin in ECRS

In current, it has yet been known that the regulatory mechanism of periostin in ECRS, but it widely acknowledged that related cytokines may affect periostin levels.

ECRS is characterized by EOS infilitration, which plays a central role in ECRS pathological progress, and it has been indicated that expression of periostin is associated with EOS [53]. It is not hard to see that eosinophilis can accelerate periostin expression.

This part mainly focused on EOS and Th2 cytokines how to regulate periostin expression. Ninomiya et al. found serum periostin level was a positive correlation with eosinophil count in peripheral bloods in ECRS patients [39]. Except for inflammatory cytokines, mediators related to remodeling also regulate secretion of periostin in ECRS. For example, there are some studies that indicate that local TGF-β and BMP (bone morphogenetic protein) could induce periostin expression by fibraoblast via depended-FAK (focal adhesion kinase) signal pathway [54], growth factors such as (fibroblast growth factors, platelet-derives growth factor and Angiotensin II) could upregulate periostin levels through PI-3K signal [55], Angiotensin II could stimulate fibroblast to secrete periostin expression via Erk1/2/TGF-β1 and Ras/p38 and MAPK/CREB signal way [56]. In ECRS patients, periostin could be expressed both in epithelial cells and fibroblast, and, TGF-β has been a key point in tissue remodeling in ECRS patients, because of which, we mainly summarize how TGF-β regulates periostin expression in ECRS.

Sidhu et al. [57] found that TGF-beta could stimulate epithelial cells and fibroblast cells to secrete periostin, importantly and Halwani et al. [58] proved that the main source of TGF-beta is eosinophils, which means eosinophils could indirectly increase periostin expressions. TGF-beta, as a positive promoter, can combine with IL-4 and IL-13 to promote levels of periostin, appealing synergy effects.

Periostin is thought to be a downstream signal of IL-4 and IL-13 in ECM through autocrine and/or paracrine ways, which in turn IL-4 and IL-13 could have an influence on levels of periostin. Takayama et al. [2] found, in mice with IL-4 or IL-13 knockout, periostin could control the extent of subepithelial fibrosis via mediating the levels of IL-4 and/or IL-13. Given previous findings in lower airway diseases, rhinologist pays more attention on how to regulate periostin in human nasal epithelial cells through IL-4/IL-13 signal pathways. Yuyama et al. [59] found that in the cultivated human epithelial cells, stimulated with IL-4 and IL-13, periostin mRNA expression levels increased compared with control.

### Periostin in diagnosis and treatment of ECRS

Different CRS subgroups have various pathological features and this difference means unique mechanism extense. If we could recognize mechanism of different endotypes CRS via characterized biomarkers, this not only could help doctors diagnose, but also guide to patients personalized treatments.

Currently, the diagnosis of ECRS mainly relies on examination of mucus tissue by pathologist, but this approach has more invasive and worse compliance, so more and more people prefer to explore more portable ways to differentiate ECRS and non-ECRS. Nowadays, clinically peripheral EOS, EOS% and *E*/*M* ratio [60, 61] always have been thought as biomarkers, but compared with above indicators, periostin has advantage on ECRS because which could reflect inflammatory severity and remodeling extent. Through a logistic research, people found that we could regard serum periostin as a better-predicted biomarker to goji pheripheal eosinophilic inflammation degree instead of eosinophilic counts, FeNO levels or serum IgE [62]. Periostin has made contibution on diagnosis, treatment and prognosis in ECRS.

For diagnosis, currently, in clinical, CRS is always treated using oral/topical steroids and surgery and antibody, however, some patients remain recalcitrant. Many researches had been underwent to find novel biomarkers to predict disease outcomes and guide individualized therapy. But, if diagnosis of ECRS only counts on periostin seems inaccurate, Xu et al. [63] suggests periostin combined with blood eosinophils and basophils might be potentially better to refine ECRS.

In treatment for ECRS, glucocorticoids (GCs) treatment including dexamethasone or tomography could suppressed periostin-caused effects [49]. In supporting of this, researches applied GCs as therapeutic agents into CRSwNP patients and achieved better outcomes through inhibiting tissue remodeling [64]. For treatment, ICSs steriod is common medicines for ECRS patients, but there still are a part of patient with bad outcomes in combination with studies [65] that ICSs tolerance was related to high serum expression of periostin and dominate eosinophils. Some people suggest applying anti-periostin into ECRS patients especially for tolerance with ICSs, which may improve clinical outcomes. Through above findings, we could make a decision that periostin may have a negative effect on ICSs for ECRS. Nagasaki et al. [66] classified asthma into eos and neutrophils asthma and found high-periostin asthma patient had bad outcomes with ICSs treatment verus low-periostin patients. Lately, some studies with ECRS patients also had similar resulting, after treating with ECRS patients, people found periostin levels became lower than before treatment [67]. We think periostin could predict sinus mucus inflammation and therapy effects and use this to choose therapies for ECRS.

As monoclonal antibody developed, some scholars aimed to evaluate the effect of monoclonal antibodies including omalizumab, mepolizumab, methylprednisolone and doxycycline on nasal and systemic periostin expression and demonstrated that monoclonal antibodies could inhibit or reduce the periostin to achieve a better outcome in some ECRS patients.

De Schryver et al. proved that methylprednisolone treatment for ECRS patients could decrease serum periostin levels at 4-week follow-up, and omalizumab-injected treatment could achieve same outcomes at 8-week follow-up, which should be connected with eosinophilic inflammation [68].

Tajiri et al. [69] through a small groups found ECRS patients with omalizumab anti after 16 weeks, these patients could improve nasal booking and oflamaotory symptoms via Lund–Mackay and SNTO-20 and serum periostin levels decreased. Regarding previous studies, lebrikizumab treatment could effectively curb exacerbation of asthma especially in patients with high serum POSTN levels, which suggests us that we need further studies to investigate whether lebrikizumab could treat ECRS patients and how outcomes differentiate among various subgroups. In the very near further, it may be possible to offer personalized medicine for ECRS patients where treatment is based on molecular biomarkers for the endotype or subendotype activated in an individual patient.

For prognosis, ECRSwNP patients have more likely to predict worse outcomes and higher risk for recurrence after surgical treatment compared with non-ECRSwNP [70] Serum periostin has been recommended as a novel biomarker for postoperative recurrence of ECRS, especially for ECRSwNP.

Zhang et al. demonstrated utility of periostin as a longitudinal biomarker for ECRS disease burden treated with endoscopic sinus surgery, through estimating periostin levels, and found elevated levels in ECRS patients underwent surgery decreased threefold after follow-up visit after surgery [67]. This reminds us that periostin expression might reflect disease activity.

Additionally, Takahiro Ninomiya et al. [39] found serum POSTN could be used for examining postoperative CRSwNP recurrence as a biomarker with the optimal cut-off point of 115.5 ng/ml according to the closet point top-left in ROC curve, which means serum POSTN could be a another candidate biomarker to predict postoperative recurrence. Fujieda et al. [71] found that ECRSwNP patients with a high serum periostin level had a positively correlation with recurrence rate, indicating that the preoperative serum periostin level might be thought as a clinical marker for recurrence after FESS surgery. This results can make more contributions to evaluating prognosis and communicating with patients, but there also is a defect lacking large population and without considering age sex territory and basic conditions to establish a more systemic standard.

Measurement of serum POSTN is simple and practical in hospital, which means we could use serum POSTN to be a biomarker for predicting the recurrence.

## Unresolved issues associated with periostin in ECRS

These findings are consistent with published literature and support evidence that increased expression of periostin is likely to be significant in the pathophysiology of ECRS. From above findings, we predict that we can reduce eosinophilic inflammation via a new viewpoint-inhibiting periostin, which has been proved to play a critical role in ECRS. However, to better apply periostin into ECRS in clinical, there still are some problems as follows.

First, periostin has played an important role in tissue remodeling and inflammation, but how this regulates globlet cells and mast cells, and the relationship between epithelial cells and perisotin still need more researches Second, epigenetic regulation in CRS has attracted more attention, which includes DNA methyl, histone and micro RNA, and epigenetic regulation also may regulate ECRS via periostin, we need to establish knockdown and/or knockout periostin in vitro models to find more underlying mechanisms. Third, nasal lavage fluid also is thought as a portable method for rhinologist in clinical, and there has been unknown with periostin expression in nasal, and whether it could be a indactor instead of serum or/and tissue producation In addition, the relationship between remodeling and inflammation in nasal epithelial has been pay more attention, combined with published studies, periostin has participated in inflammation and osteitis and mucus and bone remodeling respectively, but in the connection between them the role of peiosin plays is reminded to explore. It also should be established systematic inflammatory endotypes for ECRS in combination with various levels of periostin production to determine ECRS and predict severity of ECRS rather than traditional classification. Only depending on one indicator diagnose and treat ECRS lacks sensitivity and specificity and exploring new biomarkers in order to combine this with clinical treatment. Moreover, there still remains unclear whether nasal microbiome such as whether *S. aureus* can induce production of periostin, after all some microbiomes could influence mucosal function via enhancing related cytokines expressions. From the viewpoint of environment and lifestyles, different areas, ethnics, and with or without smoking may affect periostin expressions, to be frankly, this needs further surgery in a large population. Importantly, anti-periostin therapy still should be tested both in animals and clinical experiments.
